# Enhanced expressions of microvascular smooth muscle receptors after focal cerebral ischemia occur via the MAPK MEK/ERK pathway

**DOI:** 10.1186/1471-2202-9-85

**Published:** 2008-09-15

**Authors:** Aida Maddahi, Lars Edvinsson

**Affiliations:** 1 Division of Experimental Vascular Research, BMC A13, Lund University, 221 84 Lund, Sweden; 2 Department of Internal Medicine, Institute of Clinical Sciences, Lund University, Lund, Sweden

## Abstract

**Background:**

MEK1/2 is a serine/threonine protein that phosphorylates extracellular signal-regulated kinase (ERK1/2). Cerebral ischemia results in enhanced expression of cerebrovascular contractile receptors in the middle cerebral artery (MCA) leading to the ischemic region. Here we explored the role of the MEK/ERK pathway in receptor expression following ischemic brain injury using the specific MEK1 inhibitor U0126.

**Methods and result:**

Rats were subjected to a 2-h middle cerebral artery occlusion (MCAO) followed by reperfusion for 48-h and the ischemic area was calculated. The expression of phosphorylated ERK1/2 and Elk-1, and of endothelin ET_A _and ET_B_, angiotensin AT_1_, and 5-hydroxytryptamine 5-HT_1B _receptors were analyzed with immunohistochemistry using confocal microscopy in cerebral arteries, microvessels and in brain tissue. The expression of endothelin ET_B _receptor was analyzed by quantitative Western blot. We demonstrate that there is an increase in the number of contractile smooth muscle receptors in the MCA and in micro- vessels within the ischemic region. The enhanced expression occurs in the smooth muscle cells as verified by co-localization studies. This receptor upregulation is furthermore associated with enhanced expression of pERK1/2 and of transcription factor pElk-1 in the vascular smooth muscle cells. Blockade of transcription with the MEK1 inhibitor U0126, given at the onset of reperfusion or as late as 6 hours after the insult, reduced transcription (pERK1/2 and pElk-1), the enhanced vascular receptor expression, and attenuated the cerebral infarct and improved neurology score.

**Conclusion:**

Our results show that MCAO results in upregulation of cerebrovascular ET_B_, AT_1 _and 5-HT_1B _receptors. Blockade of this event with a MEK1 inhibitor as late as 6 h after the insult reduced the enhanced vascular receptor expression and the associated cerebral infarction.

## Background

Acute focal cerebral ischemia results in a severely ischemic core with low residual cerebral blood flow (CBF) whereas the ischemic penumbra synaptic activity is reduced while the residual CBF is enough to maintain membrane ionic gradients. The expansion of depolarized core coincides with the occurrence of spontaneous peri-infarct spreading depolarization [1]. The tissue viability threshold and its relationship to the penumbra has focused on electrical and membrane failure in brain tissue [2,3], and therefore, it has been suggested that the ischemic depolarization increases the metabolic burden, thereby exacerbates the energy deficit, and enlarges the infarct [4]. This view has by and large neglected the fact that stroke primarily is a cerebrovascular disorder. Recently, Shin and colleagues presented data that there is neurovascular vasoconstrictor coupling during the ischemic depolarization which contributes to the hemodynamic progression of damage in focal cerebral ischemia [5]. They suggest that by reducing the adverse vascular effects of tissue depolarization is a possible way the neuroprotective drugs act to reduce the tissue injury.

We have observed a rapid transcriptional upregulation of contractile endothelin-1 (ET_B _receptors), and angiotensin II AT_1 _receptors in vascular smooth muscle cells in the middle cerebral artery (MCA) leading to the ischemic region starting immediately after induction of the cerebral ischemia [6,7]. These changes result in enhanced contraction of the vasculature leading to the ischemic region, particularly because agonists for these receptor are produced in the cerebrovascular endothelium [8]. In agreement, single receptor inhibition has in the past only been found to have limited effect in reducing cerebral infarct size after focal ischemia [3]. Therefore, we hypothesize that blocking the transcriptional upregulation of endothelin, serotonin and angiotensin receptors would reduce the cerebral infarct that occurs after focal cerebral ischemia.

To test this hypothesis, an animal model of consistently inducible cerebral ischemia was used: 2 hours reversible middle cerebral artery occlusion (MCAO) followed by reperfusion for 48 hours [9]. We present the novel observations that there is upregulation of the mitogen-activated protein kinase (MAPK) extracellular signal-regulated kinase (ERK1/2), the transcription factor Elk-1, and the contractile receptors for endothelin (ET_A _and ET_B_), angiotensin II AT_1_, and 5-hydroxytryptamine 5-HT_1B _receptors in both the MCA leading to the ischemic region and in microvessels within the infarct area but not in adjacent brain tissue. Systemic treatment with the MEK1 inhibitor U0126, given at the start of the reperfusion or at 6 hours afterwards abolished the enhanced receptor protein expression and reduced the infarct volume.

## Results

### Signal transduction after MCA occlusion

**Figure 1:**
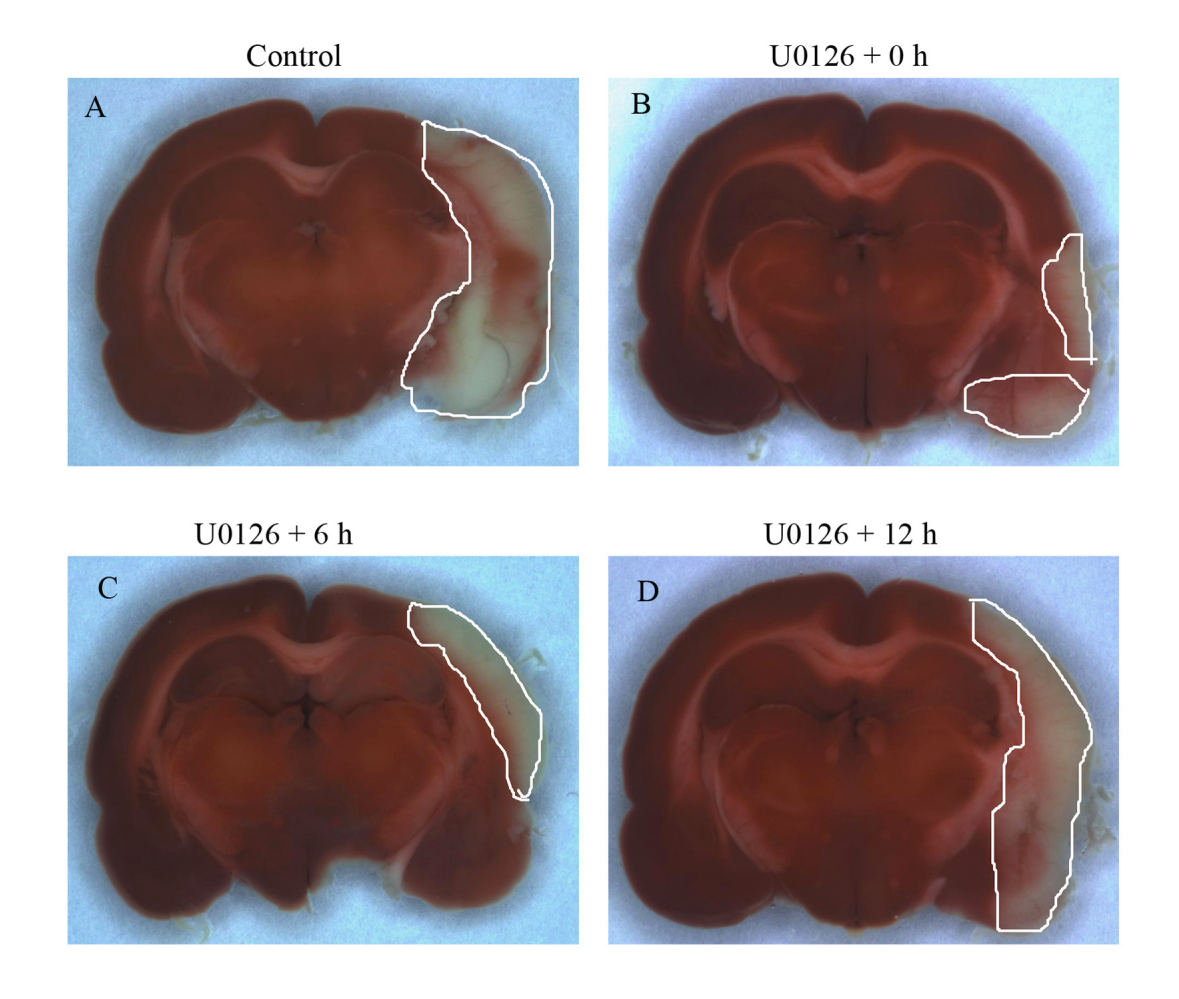
Typical examples of coronal brain sections that show smaller ischemic areas in animals treated with U0126 immediately after reperfusion after MCAO (0 h), or given at 6 and 12 hours after MCAO and 48-h reperfusion versus animals treated with vehicle (A).

**Figure 2:**
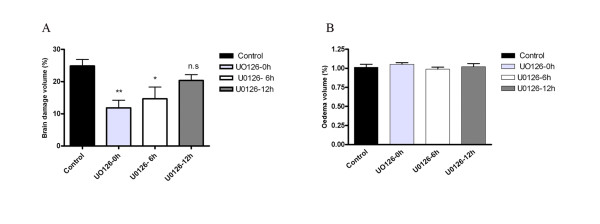
**A: **Measurements of the brain damage (% of total volume) showed a significant. Decrease in infarct size on the ischemic side in animals treated with U0126 starting at 0 h (11.8 ± 2 %**) and 6 h (14.6 ± 3 %*) after MCAO as compared to the control group (24.8 ± 2 %) and treated after 12 h (20.3 ± 1 %)(*P < 0.05 ** P < 0.01). **B: **Analysis of the edema (% of total brain volume) showing no significant change in the U0126 treated groups (0 h; 1.05 ± 0.02, 6 h; 0.98 ± 0.02 and 12 h; 1.02 ± 0.04) with the control rats (1.01 ± 0.03). Values are the mean ± s. e. m. and *n *= 6–7.

Distal MCAO resulted in an abrupt decrease in CBF over the dorsolateral cortex; flow was reduced to 15 ± 3% of the baseline flow in the ischemic region. After 2 hours the block was removed. The flow subsequently returned to baseline. The animal were carefully monitored during the following 48 hours and then sacrificed. We calculated the neurology score (MCAO: 4.0 ± 0.5, *P *< 0.05 versus sham operated animals) (Table 1), collected tissue for immunostaining and Western blot, and determination of the infarct volume (24.8 ± 1.4% of total cerebrum) and the degree of edema (Fig. 1 and 2). The physiological parameters did not differ between the groups (Table 2). There was an increase in body temperature after MCAO in all groups which are in agreement with previous studies [10]; this did not differ between the different groups in our study.

**Table 1:** Neurological Scores measured at 0, 24 and 48 hours after MCA occlusion using an established scoring scale [26].

Neurology score	Control (n = 7)	U0126 – 0 h (n = 7)	U0126 – 6 h (n = 6)	U0126 – 12 h (n = 6)
0 h	4 ± 0.0	4± 0.0	4 ± 0.0	4 ± 0.0
24 h	3.8 ± 3.4	3 ± 0.6	3.5 ± 0.5	3.8 ± 0.3
48 h	4 ± 0.5	2 ± 0.7	3.3 ± 0.7	3.8 ± 0.3

Comparison between the U0126 treated groups and vehicle treated rats (control). Data are expressed as mean ± S.D and n = number of rats.

**Table 2:** Physiological parameters

Physiological Parameters	Control (n = 7)	U0126, 0 h (n = 7)	U0126, 6 h (n = 6)	U0126,12 h (n = 6)
pO_2 _(mmHg)	97.2 ± 2	108.3 ± 3.2	107.6 ± 7	120.4 ± 10
pCO_2_(mmHg)	52.6 ± 3	50.6 ± 1.9	49.6 ± 4.5	48.5 ± 0.6
pH	7.3 ± 0.01	7.3 ± 0.01	7.3 ± 0.02	7.5 ± 0.01
Plasma glucose (mmol/L)	12.1 ± 0.4	10.2 ± 0.6	11.1 ± 0.3	10.7 ± 0.4
MAP (mmHg)	91.1 ± 2.2	92.6 ± 1.8	91.8 ± 2.5	101.4 ± 4.7
Temperature during operation (°C)	37.1 ± 0.2	37.2 ± 0.1	37.2 ± 0.1	37.3 ± 0.2
Temperature before reperfusion (°C)	39.3 ± 0.1	39.2 ± 0.1	39.3 ± 0.2	38.9 ± 0.2
Temperature after reperfusion (°C)	38.1 ± 0.1	38.6 ± 0.3	38.1 ± 0.1	38.6 ± 0.2
Weight loss 48 h after occlusion (%)	13.4 ± 1.3	8.0 ± 1.8	16.1 ± 3.2	10.3 ± 2.4

Values are the mean % of control ± s.e.m., *P *> 0.05 between the groups, n = number of rats.

**Figure 3:**
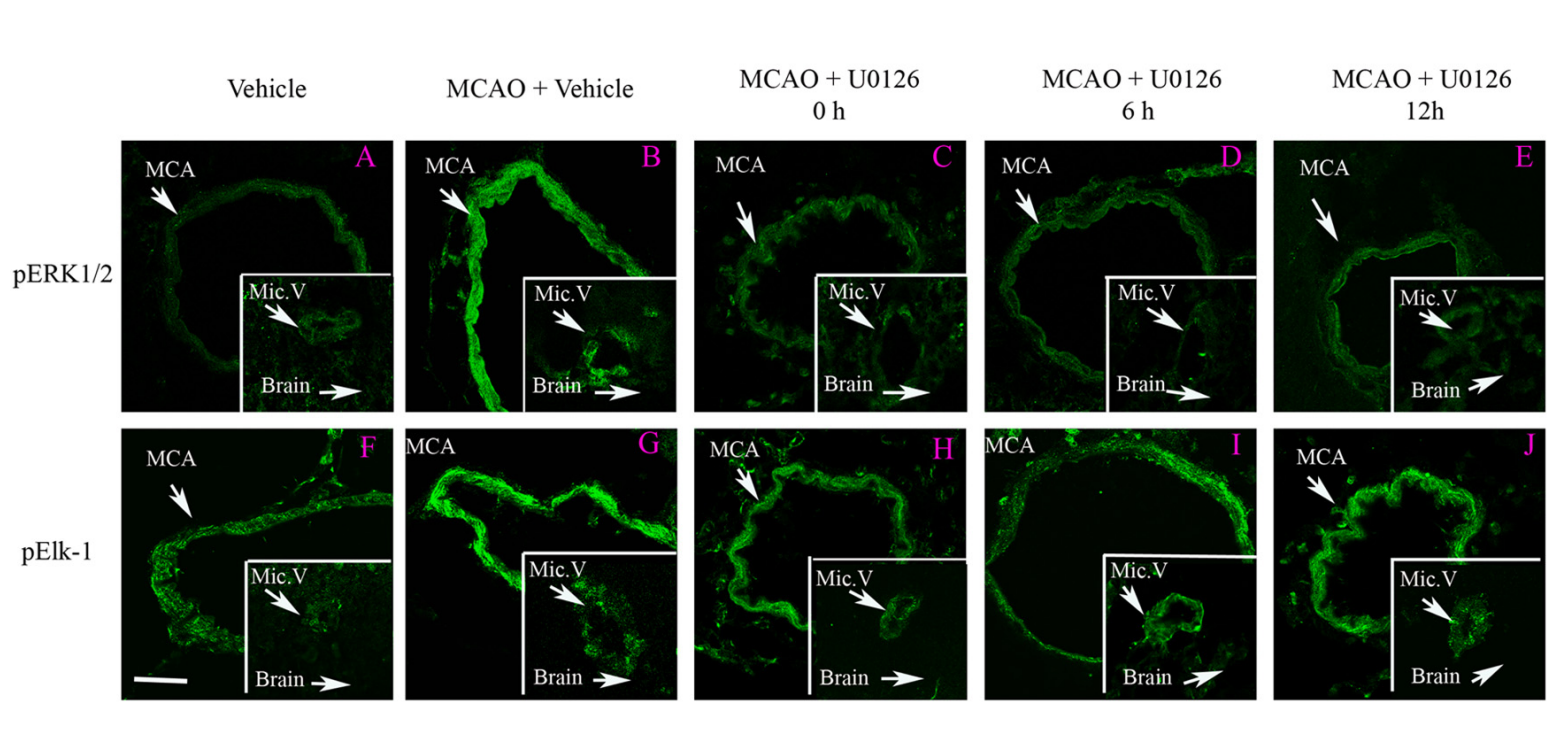
Immunofluorescence staining for pERK1/2, and transcription factor pELK-1 in the ischemic middle cerebral artery (MCA), cerebral microvessels (Mic.V), and surrounding brain tissue (Brain) in rats treated with vehicle, MCAO + vehicle, or MCAO plus U0126 at different time points. There was an increase in activated (phosphorylation = p) ERK1/2 and ELK-1 in the smooth muscle cell layer of ischemic vessels (MCA and Mic.V) as compared to vehicle. Treatment with U0126 starting at 0 h, 6 h and 12 h after occlusion prevented the increase in expression of pERK1/2 in smooth muscle cells of the MCA and Mic.V as studied at 48 h. Data were obtained with confocal microscopy. Scale bar, 50 μm.

**Figure 4:**
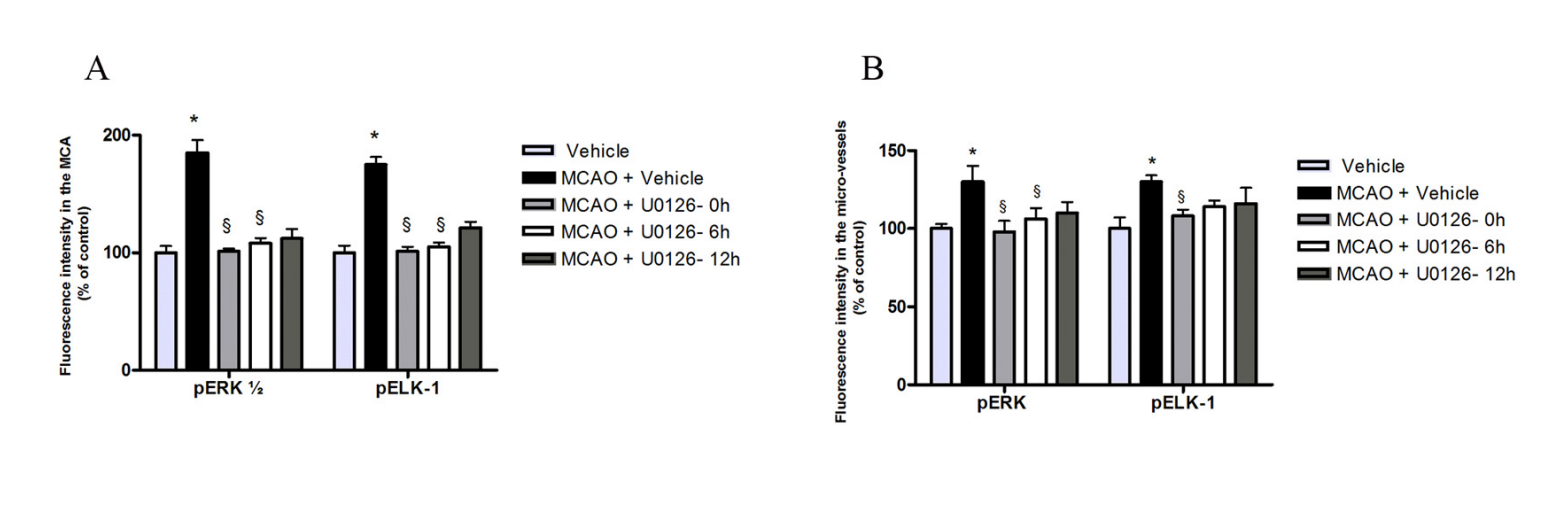
**A and B: **Bar graph demonstrating the fluorescence intensity for pERK1/2, and pElk-1 in the MCA and micro-vessels. There was a significant increase of pERK1/2, and pElk-1 protein expression in MCAO as compared to control. This was prevented with U0126 treatment starting at 0 h and 6 h but not 12 h. Results are presented as the mean percentage relative to control ± s.e.m.; *n *= 5. *P < 0.05 significant difference between control groups and MCAO, § < 0.05 significant difference between MCAO and treated groups.

**Figure 5:**
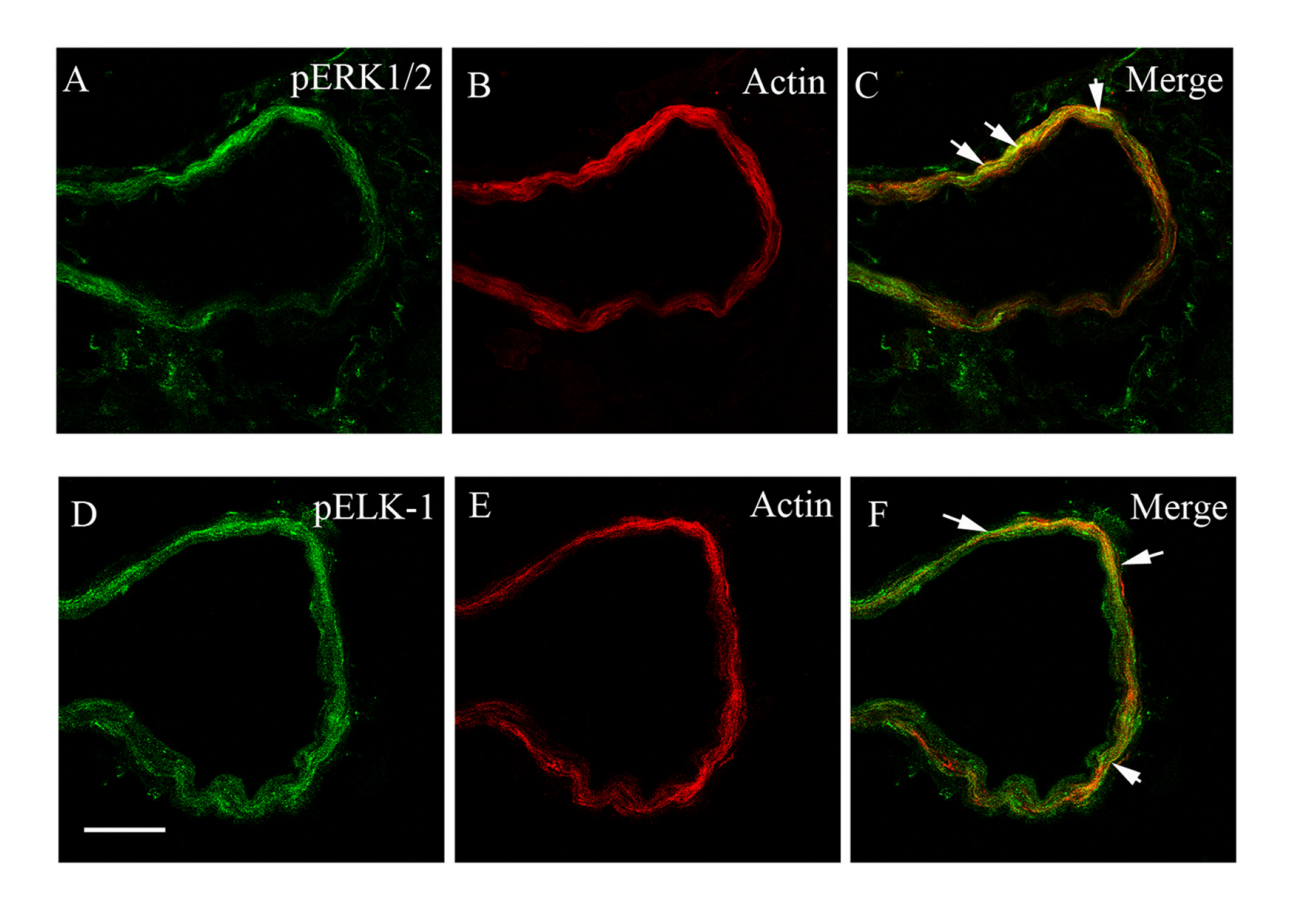
Double immunofluorescence staining for pERK1/2, pElk-1 and actin in smooth muscle cells in the middle cerebral artery after MCAO. (A) Localization of pERK1/2 immunoreactivity, (B) actin immunostaining, and (C) their co-localization in the smooth muscle cells. (D) The pElk-1 immunoreaction, (E) the actin immunostaining, and (F) their co-localization in the smooth muscle cells. The scale bar represents 50 μm.

We subsequently assessed if MCAO leads to activation of pERK1/2 and pElk-1 in the smooth muscle cells of the MCA, the associated microvessels, and in brain tissue. There was weak staining of both in vehicle control (Fig. 3). The results showed that pERK1/2 and pElk-1 were markedly activated at 48 hours after the MCAO + vehicle (Fig. 3). The pERK1/2 and pElk-1 immunoreactivity were localized in the cytoplasm of the smooth muscle cells as verified by co-localization experiments with smooth muscle specific actin (Fig. 5). As can be seen in the illustration there was a significant enhanced expression of pERK1/2 (185 ± 11 %) and pElk-1 (174 ± 6 %) in the MCA leading to the infarct and in associated microvessels (130 ± 10 % and 130 ± 6 %, respectively) (Fig. 4). However, there was no significant change in their expression in associated brain tissue (106 ± 3 % and 117 ± 13 %, respectively) or in other regions of the brain. There was a weak expression of pElk-1 in cell bodies within the brain tissue around the MCA.

### Inhibition of signal-transduction

Administration of the MEK1-specific inhibitor U0126 (30 mg/kg, intraperitoneal), which blocks the enzymatic activity of MEK1 [11] reduced both the infarct volume and the neurology score when given in conjunction with the start of the reperfusion (0 hour) or at 6 hours after the MCAO; the reductions were significant for infarct volume (11.8 ± 2 % and 14.6 ± 3 %; respectively of cerebrum, *P *< 0.05; Fig. 1 and 2A), and neurology score (2 ± 0.7 and 3.3 ± 0.7; respectively, *P *< 0.05) but not for the edema (Table 1) (Fig. 2B). The administration of U0126 with start at 12 hours after the initiation of reperfusion did not result in a significantly diminished infarct volume (20.3 ± 1 % of cerebrum; Fig. 2A) or the neurology score (3.8 ± 0.3; Table 1).

We subsequently assessed if MEK1 inhibition altered the cerebrovascular activation of pERK1/2 and pElk-1 in the vascular smooth muscle cells after MCAO. The results showed that systemic treatment with U0126 abolished the increase in pERK1/2 and pElk-1 activation after MCAO when the treatment was given in conjunction with reperfusion (0 hour) or with start 6 hours, and for pERK1/2 12 hours after the start of the reperfusion (Fig. 3 and 4). In the contralateral hemisphere there was only weak pERK1/2 and pElk-1 activity at baseline in the vasculature, and this was not affected by MCAO (Fig. 3); the levels of activity were comparable to those of the vehicle control. There was no significant change in brain tissue for pERK1/2 and pELK-1 activity (Fig. 3).

### Cerebrovascular receptor expression

**Figure 6:**
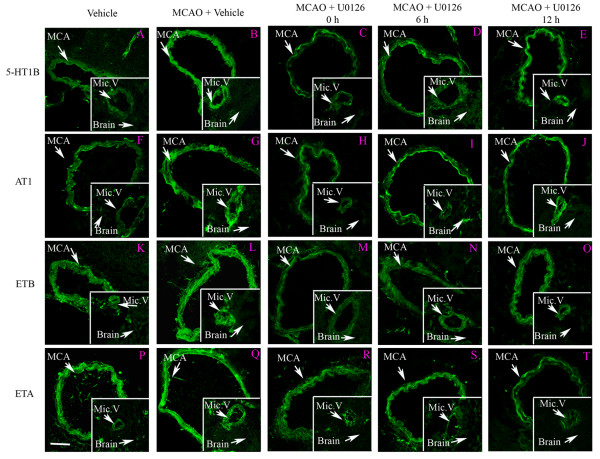
Immunofluorescence staining for 5-HT_1B_, (A-E), AT_1_(F-J), ET_B _(K-O), and ET_A _(P-T) receptors in the ischemic middle cerebral artery (MCA), cerebral microvessels (Mic.V), and surrounding brain tissue (Brain). There was a clear increase in 5-HT_1B_, AT_1_, and ET_B _receptor protein levels in the smooth muscle cell layer of ischemic vessels (MCA and Mic.V) as compared to vehicle control. Treatment with U0126 starting at 6 h after MCAO prevented the increase in expression of 5-HT_1B_, AT_1 _and ET_B _receptors in smooth muscle cells of the MCA and Mic.V but not at 12 h. The ET_A _receptor is normally expressed fairly strong, and expression is not upregulated in ischemia. U0126 treatment did not change the expression of ET_A _receptor protein in the smooth muscle cell layer. There was no significant difference in expression of receptor protein levels in control brain tissue, in ischemic brain tissue, and tissue from animals treated with U0126. Data were obtained with confocal microscopy. Scale bar, 50 μm.

**Figure 7:**
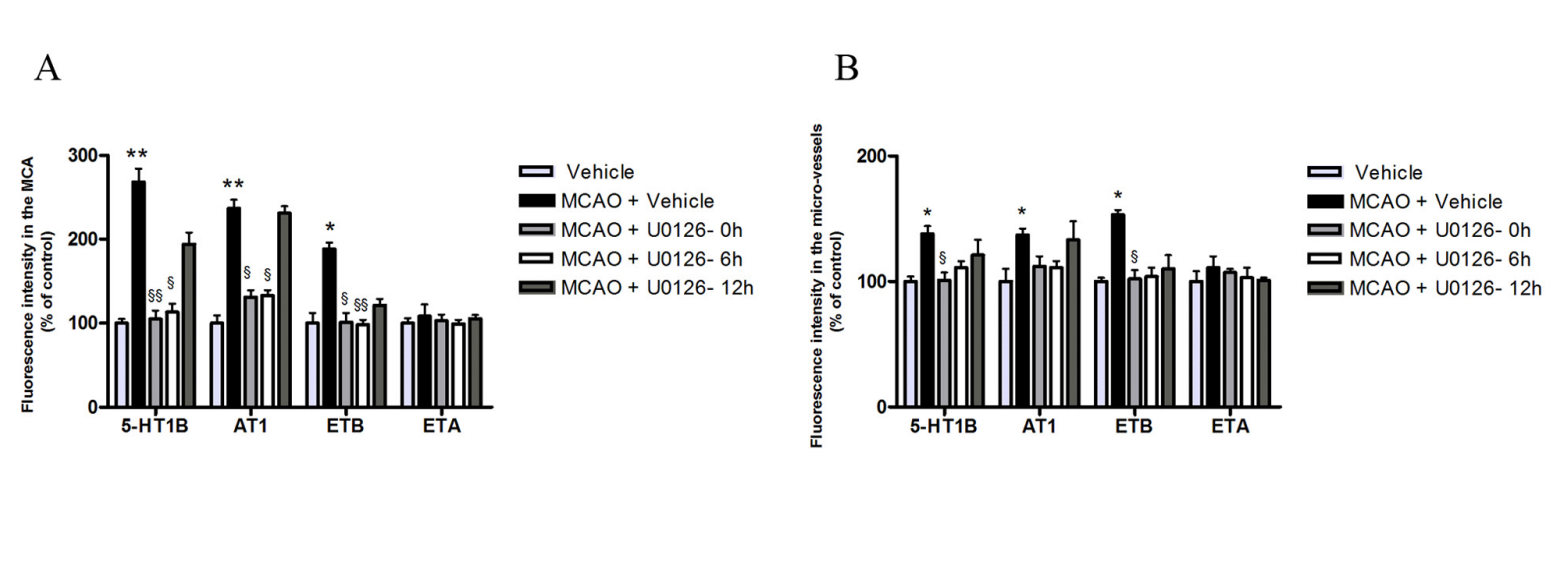
**A and B: **Bar graph demonstrating the fluorescence intensity for 5-HT_1B_, AT_1_, ET_B _and ET_A _receptors in the middle cerebral artery and micro-vessels. There was a significant increase of 5-HT_1B_, AT_1_, and ET_B _protein expression in MCAO as compared to control. This was prevented with U0126 treatment starting at 0 h and 6 h in the MCA and in the micro-vessels but not 12 h. Results are presented as the mean percentage relative to control ± s.e.m.; *n *= 5. *P < 0.05, **P < 0.01. Statistically significant differences between the groups are shown; §< 0.05, §§ < 0.01 significant difference between MCAO and treated groups.

**Figure 8:**
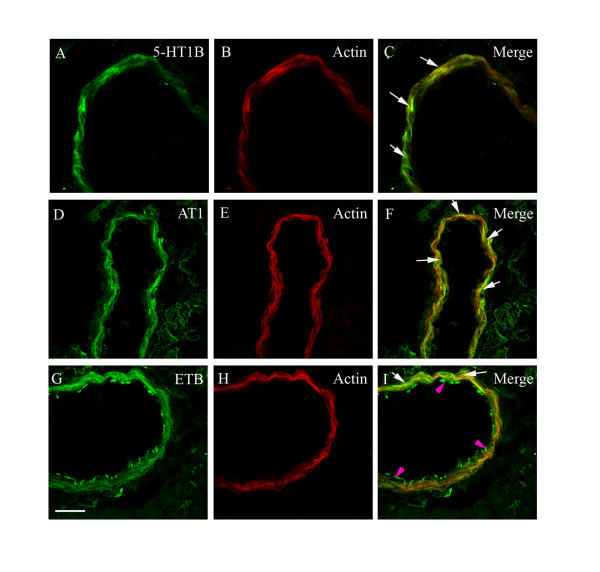
Co-localization of the immunofluorescence for 5-HT_1B _(A, C), AT_1 _(D, F) and ET_B _(G, I) receptor proteins and smooth muscle cell actin (B, E, H) in the MCA after MCAO. Their co-localization is depicted in the merged pictures to the right (C, F, I). Scale bar, 50 μm.

To investigate whether the MAPK activity leads to changes in receptor protein transcription, we analyzed the expression of vascular receptors using immunostaining and image analysis with confocal microscopy. We observed for the first time a significant increase in the expression of endothelin ET_B_, angiotensin AT_1_, and 5-hydroxytryptamine 5-HT_1B _receptors in smooth muscle cells not only in the ischemic MCA but also in smooth muscle cells of cerebral microvessels associated with the ischemic region (Fig. 6 and 7). There were no changes in receptor expression in contralateral vessels. Importantly, the endothelin ET_A _receptor expression was unchanged after MCAO and following treatment at all locations (Fig. 6 and 7). Double immunostaining for endothelin ET_B_, angiotensin AT_1_, and 5-hydroxytryptamine 5-HT_1B _receptors versus smooth muscle actin, expressed in the smooth muscle cells, revealed clear co-localization. All three receptors co-localized with the smooth muscle cells, in addition, endothelin ET_B _receptor protein was located in the endothelial cells of the cerebral vessels (Fig. 8); previous studies with the endothelial marker CD31 has verified this in cerebral arteries [12]. MCAO did however not show enhanced (or altered) expression of the endothelial ET_B _receptors.

Systemic treatment with the MEK1 inhibitor abolished the increase in receptor expression in both vascular regions when treatment was initiated either at reperfusion (0 hour) or 6 hours afterwards but not when starting 12 hours after reperfusion (Fig. 6); this correlates well with the reduction in infarct volume at the same time points (Fig. 1 and 2A).

### Western blot

**Figure 9:**
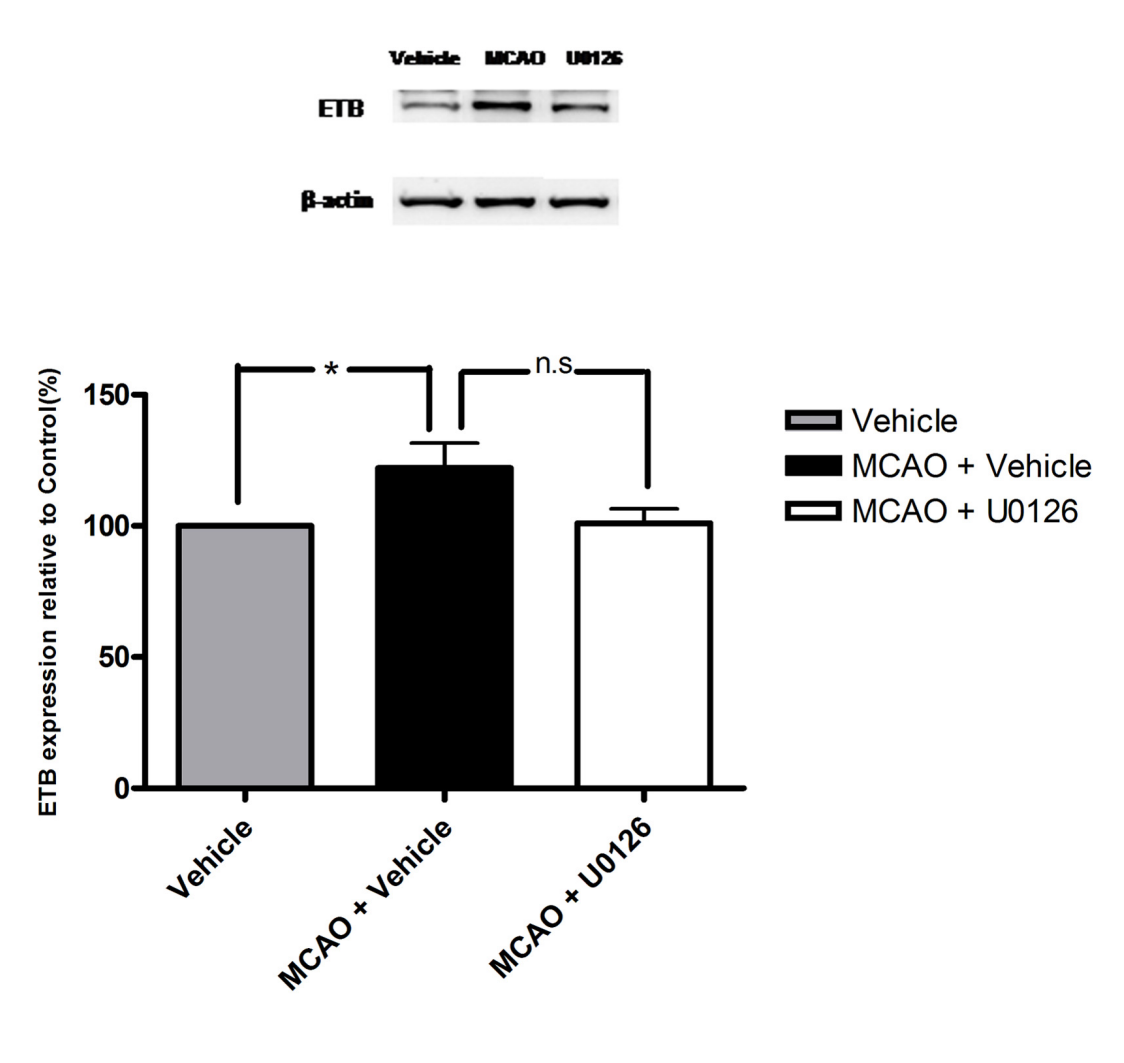
Expression and upregulation of ET_B _protein levels in middle cerebral artery after 48 h of MCAO with β-actin as a loading control is shown above. Treatment with U0126 decreased the enhanced expression of ET_B _receptor protein after MCAO. Data is expressed as mean ± s.e.m., n = 4, *P < 0.05.

The Western blot experiments showed a significant increase in ET_B _protein level after MCAO as compare to the vehicle group (122 ± 9 %; p < 0.05). Treatment with the MEK inhibitor (U0126) given in conjunction with reperfusion (0 hour) prevented the increase in the ET_B _receptor protein (101 ± 5%) (Fig. 9).

## Discussion and conclusion

We have observed that acute cerebral infarction followed by reperfusion in the rat is accompanied by upregulation of the functional contractile phenotype and the mRNA expression of endothelin ET_B _and angiotensin AT_1 _receptors [6,7]. In the present study we show for the first time that the proteins of the contractile receptors ET_B_, AT_1 _and 5-HT_1B _receptors are upregulated in the smooth muscle cells of the MCA leading to the ischemic region and in microvessels associated with the focal ischemia. The co-localization studies verified that the enhanced expression is located in the smooth muscle cells. We have previously shown with confocal microscopy and double immunostaining that the enhanced receptor expression is localized to the smooth muscle cells (co-localize with smooth muscle actin) and not to the adventitia or the endothelial cells (with CD31 staining) in conjunction with experimental subarachnoid hemorrhage [12]. The enhance receptor expression was verified by Western blot of the ET_B _receptor protein.

This upregulation was associated with activation of the signal transduction proteins pERK1/2 and the transcription factor pElk-1. Administration of the MEK1 inhibitor U0126, acting upstream of ERK1/2, abolished their activation as well as the enhanced receptor expression, and reduced the infarct volume. Importantly this reversal worked if U0126 was given both immediately following the reperfusion (0 hour) and at 6 hours after the insult. If the MEK1 inhibitor was given 12 hours after the start of the reperfusion there were no significant changes in infarct volume, neurology score or receptor expression but still the MAPK pERK1/2 and the transcription factor pElk-1 levels were depressed. The reduction in infarct volume occurred in concert with a reduction in receptor expression and activation of ERK1/2 and Elk-1 in the middle cerebral artery and in microvessels on the ischemic side of the brain. This agrees with a previous study that showed a transient (within the first hours) increase in the MEK/ERK pathway located to neurons and astrocytes in conjunction with focal ischemia (but not studied before in cerebral vasculature); systemic administration of U0126 inhibited this response [13]. We suggest that the MEK/ERK pathway participates as a switch-on mechanism for the receptor upregulation and the associated brain damage. In support, we found in a Western blot time study of global ischemia that there was early activation of this pathway already within one hour [14]. In addition, there was no activation of c-jun N-terminal kinase (JNK) or p38 at time points before 24 hours [14]. The MEK/ERK pathway uses several transcription factors, the pElk-1 studied here can be regarded as an example of one of those. We suggest that there is a "switch-on" mechanism that is accessible to early antagonism but if therapy is give after this (as at 12 hours after reperfusion) it will be too late to modify the outcome.

One might suggest that another way to reduce the infarct would be to use specific endothelin-receptor [15] or angiotensin-receptor antagonists [16]; there are data to provide support for such a concept. Dual blockade of the AT_1 _and ET_A _receptors produced stronger reduction in infarct than single receptor blockade; this is due to upregulation of several receptor subtypes after MCAO [17]. We have in this study demonstrated that multiple receptor subtypes are upregulated in the vasculature following the ischemia (at least 3 subtypes as shown in this study); we propose that blockade at the transcriptional/translation level is a superior approach to specific receptor antagonism and avoids significant systemic effects and at the same time modulates the enhanced expression of several receptors.

It is intriguing that U0126 does not affect phosphorylation of p38 or JNK in cultured neurons [18], *in vivo *in brain tissue [13] or in cerebrovascular smooth muscle cells [14]. These findings enable us to exclude the possibility that U0126 reduced the infarct volume by nonspecific inhibition of pro apoptotic mechanisms. In addition, U0126 increases phosphorylation of MEK1/2 in cortical neurons; hence, U0126 does not affect upstream components of MEK1/2 [19]. Thus, it is reasonable to assume that the effect of U0126 is due to inhibition of cerebrovascular MEK1 activity. Treatment with U0126 abolished the increase in receptor expression both in the MCA and in microvessels without affecting activity in contralateral blood vessels or in the adjacent brain tissue. This is consistent with previous reports that administration of U0126 in conjunction with MCAO decreases pERK1/2 immunoreactivity in the ischemic brain region of the mouse [18] and rat [20]. In the mouse study, 3-h MCAO was followed by reperfusion for 24 h, but U0126 reduced the infarct volume only when administered in conjunction with MCAO [18]. Another selective MEK1 inhibitor PD98059 failed to protect ischemic cell death in the CA1 region in the gerbil [21]. It revealed however a neuroprotective effect when given intracerebroventricular [22]. The MEK1 inhibitor SL327 reduced infarct size and improved neurological function after ischemic injury in mice [23]. The differences between the studies may be due to dose administered, the experimental model used for cerebral ischemia, and the ability of the drug to penetrate across the blood-brain barrier [13]. Here we show for the first time that U0126 is effective in a clinically relevant time frame. It was effective not only when given in conjunction with the MCAO but also 6-h after reperfusion and repeated at 24 h. In agreement with these observations, ERK1/2 inhibition does not alter cortical blood flow or alters the vessel tone in the first hour of its administration [18,24]. Hence, it is not acting through a direct vasodilator mechanism or acts acutely to interact with excitation-contraction coupling in the smooth muscle cells. Quantitative studies of regional cerebral blood flow have revealed that flow during the first hours after start of reperfusion returns to near normal levels but still there is the appearance of marked cell death putatively due to metabolic dysfunction [9]. The present data suggest that a vascular component might contribute to the development of tissue damage.

Our observations are in agreement with the diminished activity of the downstream MAPK MEK/ERK pathway and its transcription factor Elk-1, as well as the reduced expression of specific vascular receptor proteins both in large cerebral vessels and in microvessels. In functional studies there is reduced contraction following specific ET_B _and AT_1 _receptor stimulation [[6,7], and [20]]. Inhibition of this sequence of events is accompanied by reduction of neuronal death and improvement in neurology score in the same animal and agrees well with recent data on experimental subarachnoid hemorrhage[25].

We suggest that more focus should be directed towards the cerebral vasculature. Although the MEK/ERK pathway plays a crucial role in brain injury after ischemia and reperfusion, we provide here the first direct evidence of an associated vascular mechanism, involving both large cerebral arteries and brain microvessels. This may provide a novel way to search for agents that will alleviate brain injury following cerebral ischemia.

## Methods

### Middle cerebral artery occlusion

Male Wistar-Hanover rats (Møllegaard Breeding Centre, Copenhagen, Denmark) weighing approximately 300–350 g were used. Experimental procedures were approved by the Lund University Animal Ethics Committee (M43-07). The animals were initially anaesthetized using 4 % halothane in N_2_O:O_2 _(70 %:30 %). Thereafter the rats were kept anaesthetized through a mask with 2 – 2.5 % halothane during the operation. To confirm a proper occlusion of the proximal middle cerebral artery (MCA), a laser Doppler probe (Perimed, Järfälla, Sweden) was fixed on the skull (1 mm posterior to bregma and 6 mm from the midline of the right side), measuring regional cortical blood flow. A polyethylene catheter was inserted into a tail artery for measurement of mean arterial blood pressure (MAP), pH, pO_2_, pCO_2_, and plasma glucose. A rectal temperature probe connected to a homeothermal blanket was used to maintain body temperature at 37°C during the operational procedure.

An intraluminal filament technique was used to induce transient MCAO as outlined before [9]. Briefly, an incision was made in the midline of the neck, and the right common, external, and internal carotid arteries were exposed. The common and external carotid arteries were permanently ligated by sutures. A filament was inserted into the internal carotid artery via an incision in the common carotid artery and further advanced until the rounded tip reached the entrance of the right MCA. The resulting occlusion was visible by laser-Doppler as an abrupt reduction of cerebral blood flow of approximately 80–90 %. The anesthesia was then discontinued and the animals allowed recovering.

Two hours after the occlusion, the rat was re-anesthetized to allow for withdrawal of the filament and subsequent reperfusion. In conjunction with the reperfusion (0 h), at 6 h or at 12 h afterwards, and at 24 h after the start of the reperfusion in the same animal, the rats were injected intraperitoneal with 30 mg/kg U0126 dissolved in dimethylsulfoxide (DMSO) while control groups received the same volume of vehicle (DMSO). In preliminary experiments we evaluated U0126 doses varying between 10 mg/kg and 100 mg/kg (n = 3–6) (unpublished data); 30 mg/kg was the lowest that elicited a clear significant effect on infarct volume. Control rats were injected with an equal volume of DMSO at the same time points. The rats were neurologically examined immediately before sacrifice according to an established system [26]. For details of the neurology scale see [20]. Forty-eight hours after MCAO, rats were anesthetized and decapitated; the brain was removed and placed in cold buffer solution. The right and left MCAs and associated brain tissue were dissected, snap frozen in cold isopentane, and maintained at -80°C for further examination with immunohistochemistry.

The specificity of U0126 has been tested in numerous studies previously [13,18] on isolated cells and in vivo; we have performed this on cerebral blood vessels with and without the MEK1 inhibitor [20,27]. In addition, MCAO and organ culture elicited an early increase in the pERK1/2 activity but not in pP38 or in pJNK [28].

### Evaluation of brain damage and edema

Brains were sliced coronal into 2-mm thick sections and stained by 1% 2, 3, 5-triphenyltetrazolium chloride dissolved in buffer solution at 37°C for 20 min. The size of ischemic brain damage and the degree of edema (swelling of the right part of the brain relative to the left contra lateral side) were calculated as a percentage of the total brain volume using the software program Brain Damage Calculator 1.1 (MB Teknikkonsult, Lund, Sweden).

### Neurological examination

The animals were examined neurologically before recirculation and immediately before they were sacrificed, 48 hours after MCAO according to an established scoring system [26,29].

### Immunostaining

For immunostaining, the middle cerebral artery and the surrounding brain tissue were dissected, placed on Tissue TEK (Gibo, Invitrogen A/S, Taastrup, Denmark), and frozen on dry ice [17]. Thereafter, they were sectioned into 10 μm-thick slices. Cryostat sections of the arteries and tissue were fixed for 10 min in ice-cold acetone (-20°C) and rehydrated in PBST (phosphate buffer solution containing 0.3% Triton X-100) for 15 min at room temperature. The tissues were then permeabilized and blocked for 1 h in blocking solution containing PBS, 0.3% TritonX-100, 1% bovine serum albumin (BSA), and 5% normal donkey serum to ensure antibody specificity. They were incubated overnight at 4°C with primary antibodies: rabbit antihuman ET_B _(Abcam, ab1921) 1:400, rabbit antihuman AT_1 _(Santa Cruz, sc-1173), goat antihuman 5-HT_1B _(Santa Cruz, sc1460) and goat antihuman ET_A _(Santa Cruz, sc-21194) 1:100, rabbit antiphospho ERK 1/2 MAPK (Cellsignaling #4376) 1:50, and rabbit anti phospho Elk-1 (Cellsignaling #9181) 1:50 (diluted in PBS containing 0.3% Triton X-100, 1% BSA, and 2% normal donkey serum). Sections were subsequently incubated for 1 h at room temperature with secondary antibodies: Cy™^2^-conjugated donkey anti rabbit (JacksonImmunoResearch, 711-165-152) and Cy™^2^-conjugated donkey anti goat (JacksonImmunoResearch, 705-225-003) diluted 1:200 in PBS containing 0.3% Triton X-100 and 1% BSA. The sections were washed with PBS and mounted with Permafluor mounting medium (Beckman Coulter, PN IM0752). Immunoreactivity was visualized and photographed with a Nikon confocal microscope (EZ-c1, German) at the appropriate wavelength. The same procedure was used for the negative controls, but primary antibodies were omitted. The fluorescence intensity was measured with the software image J  The fluorescence was measured in 4–6 areas in each tissue (in a blinded fashion), and the mean value was used.

### Double immunostaining

Double immunostaining was done for endothelin ET_B_, angiotensin AT_1_, and 5-hydroxytryptamine 5-HT_1B _receptor protein, and phosphorylated ERK1/2 and Elk-1 proteins versus smooth muscle actin, expressed in the smooth muscle cells. The same antibodies were used as above but in addition mouse anti rat smooth muscle actin antibodies (Santa Crus, SC-53015) 1:200, diluted in PBS containing 0.3 % Triton X-100, 1 % BSA and 2 % normal donkey serum. The secondary antibodies used were donkey α-rabbit Cy™^2^- (JacksonImmunoResearch,) 1:200, donkey α-goat Cy™^2^- (Jacksson,) 1:200 and donkey α-mouse Texas Red (JacksonImmunoResearch,) 1:250 diluted in PBS containing 3 % Triton X-100 and 1 % BSA. The antibodies were then detected at the appropriate wave lengths in a confocal microscope (EZ-cl, Germany).

### Western blot experiments

The proximal MCA segments (n = 12 in each group) were harvested and frozen in liquid nitrogen and homogenized in cell extract denaturing buffer (BioSource, USA) with addition of a phosphates inhibitor cocktail and protease inhibitor cocktail (Sigma, USA). Whole cell lysates were sonicated for 2 min on ice, centrifuged at 15,000 × g at 4°C for 30 min, and the supernatants were collected as protein samples. The Protein concentrations were determined using the protein assay reagents (Bio-Rad, Hercules, CA, USA) and stored at -80°C until immunoblotting assay. The protein homogenates were diluted 1:1 (v/v) with 2 × SDS sample buffer (Bio-Rad). 25–50 μg of total proteins were boiled for 10 min in SDS sample buffer and separated by 4–15 % SDS Ready Gel Precast Gels (Bio-Rad, USA) for 120 min at 100 v, and transferred electrophoretically to nitrocellulose membranes (Bio-Rad) at 100 v for 60 min. The Membrane was then blocked for 1 h at room temperature with phosphate buffered saline (PBS) containing 0.1 % Tween-20 (Sigma) and 5 % non-fat dried milk, and incubated with primary antibody, Rabbit polyclonal to endothelin B receptor (ab 1921), diluted 1:200 overnight at 4°C, followed by incubation with anti-rabbit IgG, horseradish peroxidase (HRP)-conjugated secondary antibodies (Amersham Biosciences, Piscataway, NJ, USA) diluted 1: 5000–10.000 for 1 h at room temperature. The probed proteins were developed by .LumiSensor Chemiluminescent HRP Substrate kit (GenScript Corp., Piscataway, NJ, USA). To detect multiple signals using a single membrane, the membrane was incubated for 5–15 min at room temperature with restore plus Western blot stripping buffer (Pierce Biotechnology, Inc., Rockford, IL. USA). The membranes were visualized using a Fujifilm LAS-1000 Luminiscent Image Analyzer (Stamford, CT, USA.). The quantification of band intensity was analyzed with Image Gauge Ver. 4.0 (Fuji Photo Film Co., LTD., Japan). Three independent experiments were performed in duplicate.

### Statistical analysis

Data are expressed as the mean ± s.e.m. Statistical analyses were performed using the nonparametric Kruskal-Wallis test with Dunn's post hoc test for quantitative immunohistochemistry and one-way ANOVA analysis of variance with Dunnett's test for infarct and edema brain evaluation. For Western blot comparisons between two groups were performed using two-tailed unpaired Student's t-test and at least 3 different samples or independent experiments were analyzed in each group. *P*-values less than 0.05 were considered significant, n = number of rats.

## Authors' contributions

AM carried out the experiments, participated in design, statistical analysis and writing of the manuscript. LE conceived of the study, participated in its design, coordination and writing. All authors read and approved the final manuscript.
